# Long QT molecular autopsy in sudden unexplained death in the young (1-40 years old): Lessons learnt from an eight year experience in New Zealand

**DOI:** 10.1371/journal.pone.0196078

**Published:** 2018-04-19

**Authors:** Luciana Marcondes, Jackie Crawford, Nikki Earle, Warren Smith, Ian Hayes, Paul Morrow, Tom Donoghue, Amanda Graham, Donald Love, Jonathan R. Skinner, on behalf of the Cardiac Inherited Disease Group New Zealand

**Affiliations:** 1 Greenlane Paediatric and Congenital Cardiac Services, Starship Children’s Hospital, Auckland, New Zealand; 2 Department of Medicine, Faculty of Medical and Health Sciences, University of Auckland, Auckland, New Zealand; 3 Greenlane Cardiovascular Services, Auckland City Hospital, Auckland, New Zealand; 4 Genetics, Northern Regional Genetics Service, Auckland, New Zealand; 5 Department of Forensic Pathology, LabPlus, Auckland City Hospital, Auckland, New Zealand; 6 Department of Cardiology, Wellington Hospital, Wellington, New Zealand; 7 Department of Cardiology, Waikato Hospital, Hamilton, New Zealand; 8 Diagnostic Genetics, LabPlus, Auckland City Hospital, Auckland, New Zealand; University of Tampere, FINLAND

## Abstract

**Background:**

To review long QT syndrome molecular autopsy results in sudden unexplained death in young (SUDY) between 2006 and 2013 in New Zealand.

**Methods:**

Audit of the LQTS molecular autopsy results, cardiac investigations and family screening data from gene-positive families.

**Results:**

During the study period, 365 SUDY cases were referred for molecular autopsy. 128 cases (35%) underwent LQTS genetic testing. 31 likely pathogenic variants were identified in 27 cases (21%); *SCN5A* (14/31, 45%), *KCNH2* (7/31, 22%), *KCNQ1* (4/31, 13%), *KCNE2* (3/31, 10%), *KCNE1* (2/31, 7%), *KCNJ2* (1/31, 3%). Thirteen variants (13/128, 10%) were ultimately classified as pathogenic. Most deaths (63%) occurred during sleep. Gene variant carriage was more likely with a positive medical history (mostly seizures, 63% vs 36%, p = 0.01), amongst females (36% vs 12%, p = 0.001) and whites more than Maori (31% vs 0, p = 0.0009). Children 1–12 years were more likely to be gene-positive (33% vs 14%, p = 0.02). Family screening identified 42 gene-positive relatives, 18 with definitive phenotypic expression of LQTS/Brugada. 76% of the variants were maternally inherited (p = 0.007). Further family investigations and research now support pathogenicity of the variant in 13/27 (48%) of gene-positive cases.

**Conclusion:**

In New Zealand, variants in *SCN5A* and *KCNH2*, with maternal inheritance, predominate. A rare variant in LQTS genes is more likely in whites rather than Maori, females, children 1–12 years and those with a positive personal and family history of seizures, syncope or SUDY. Family screening supported the diagnosis in a third of the cases. The changing classification of variants creates a significant challenge.

## Introduction

Sudden unexplained death in the young (SUDY) has devastating psychological and social effects in the community and in the surviving family members. Standard forensic examination fails to identify a cause in up to half of the cases, which can sometimes be attributed to an underlying inherited arrhythmia. [1, 2]

Cardiac ion channelopathies such as long QT syndrome (LQTS), catecholaminergic polymorphic tachycardia (CPVT) and Brugada syndrome can have their first presentation as SUDY. Death can be prevented; the challenge is to identify pre-symptomatic individuals in the community. Since these conditions are usually familial, their detection amongst the deceased potentially allows family members to be identified and protected. The hallmark studies of Behr *et al* and Tan *et al* [3, 4] demonstrated that cardiac investigation of family members can lead to a diagnosis in the deceased in up to 40% of cases. Another approach is the use of genetic testing from the decedent’s DNA, the so called “molecular autopsy”. [5]

In New Zealand, a combined approach of these two methods was adopted in 2006. Sequencing of LQTS genes 1, 2, 3 (*SCN5A*, also associated with Brugada syndrome), 5, 6, and 7 was offered free of charge to the National Forensic Service in SUDY cases (age 1–40 years). Results of the first 26 months of analysis showed that molecular autopsy identified mutations and rare variants in LQTS/Brugada genes in 15% of the individuals in whom no diagnosis had been made using standard assessment. [6] Our group was pleased to be part of the largest study yet of sudden death in the young, from Australia and New Zealand, showing that with a widened molecular autopsy, up to 27% of unexplained deaths are associated with “clinically actionable” genetic variants, in a large number of genes. [7] It will take some years before we know for sure whether these have wide family significance, or are truly pathogenic by co-segregation. With continuation of the clinical service in New Zealand for a total of 8 years, and a further two years follow up, we aim with this study to focus on the long QT molecular autopsy and assess its diagnostic yield and the impact on family screening both in terms of the value to the families, and its effect on the final interpretation of the genetic variants we identified.

## Methods

New Zealand has an established inherited heart disease and sudden death registry with ethical approval from the New Zealand Health and Disability Ethics Committee (HDEC reference AKX/02/00/107/AM03). This is a signed consent-based registry where patients or legal guardians of patients under 18 and/or their next of kin gave approval for research into their cardiac condition and consented to the publication of anonymised clinical and genetic data. Since this is an audit from that registry HDEC waived the requirement for ethical approval for the present study. The report has also been approved by the chief coroner for New Zealand. [6, 8] A coordinated network of clinicians and scientists known as the Cardiac Inherited Disease Group (CIDG) provides clinical and diagnostic genetic services throughout New Zealand for families with inherited heart disease. We reviewed all cases of autopsy-negative SUDY who have undergone genetic testing for LQTS/Brugada since the beginning of the work in 2006 until December 2013. Funding for testing prior to 2008 was from charitable support, and after that was government funded via the Ministry of Justice. Previously reported cases are included in our analysis. [6] SUDY was defined as an unexpected fatal event in an apparently healthy subject or whose disease was not expected to be the cause of death, having occurred between the ages of 1 and 40 years-old.

### Molecular autopsy and assessment of pathogenicity

Genetic testing of the LQTS genes linked to types 1, 2, 3 (*SCN5A*, also associated with Brugada syndrome), 5, 6 and 7 was performed as previously described by our group and by collaborating clinical service laboratories. [6, 9] Since 2007, screening for rarer deletions and duplications by using multiplex ligation-dependent probe amplification initially and later by array comparative hybridization was included in the analysis and performed in cases where no mutations have been detected by initial sequencing. [10] Genetic variants in the LQTS/Brugada genes were assessed for potential pathogenicity by using a multifaceted approach involving evidence from the literature and mutation databases, *in silico* analysis with predictive bioinformatics programs PolyPhen 2 [11] and SIFT, [12] *in vitro* functional analyses when possible and familial cascade screening. Cases that were found to have an established or potentially pathogenic rare variant using the tools described above were selected. During the timeframe of the study, some of the genetic variants were initially described as potentially pathogenic by our group based on the information available at the time, but with ongoing investigations locally and overseas, and family screening pathogenicity of some variants came into question and were down-graded. All cases were reviewed by CIDG and the conclusion as whether the SUDY case was a consequence of LQTS/Brugada syndrome was described as follows:

1. Highly probable and probable: there was enough evidence based on clear pathogenicity of the genetic variant, circumstances of death, previous medical history and family history, including screening and co-segregation analysis to support the channelopathy as the cause of death.
2. Possible: pathogenicity of the rare variant was detected by one or more methods described above and circumstances of death were compatible, but there was not enough evidence from previous medical history, family history and family screening.
3. Uncertain: pathogenicity of the novel genetic variant has not been demonstrated with the methods described above and family screening was uninformative or is still ongoing.
4. Unlikely: there was no clear evidence of pathogenicity using the methods described above or the variant was later found to be a common polymorphism.

Family screening was defined as supportive, partially supportive or not supportive of the diagnosis of a putative channelopathy depending on the results of co-segregation analysis.

## Results

### Comparison between gene-positive and gene-negative groups

**Fig 1:**
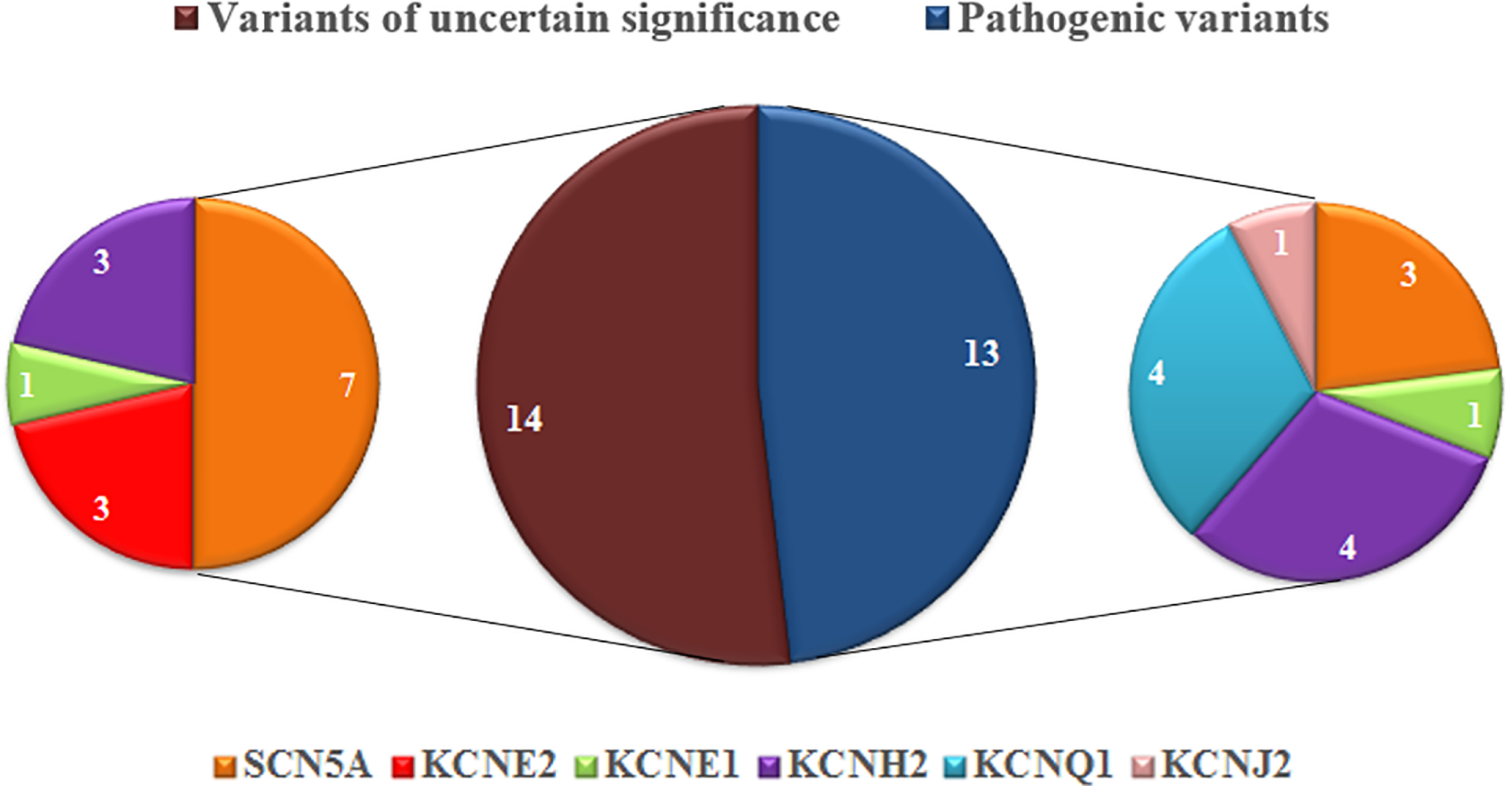
Distribution of pathogenic variants and variants of uncertain significance.

From September 2006 until December 2013, 365 SUDY cases were referred for autopsy. Amongst them, 128 (76 males, 59%) were autopsy-negative and therefore referred for molecular autopsy. A total of 31 genetic variants was found in 27 cases (21%), with 9/76 (12%) males and 19/52 (36%) females having a positive genetic test (p = 0.001). Four males had two genetic variants each. In 13/27 (48%) of the gene-positive cases the genetic variants were deemed pathogenic (i.e.: 13/128 (10%) of all SUDY cases) and the consequent channelopathy highly probably or probably the responsible for the subject’s demise–we classified these cases as “final gene-positives” for distinction. In the other 14 cases, current investigations have not yet been able to ascertain whether the variant and consequent channel dysfunction was the cause of death. In addition, some of the variants initially classified by CIDG as likely pathogenic were later down-graded to variants of uncertain significance or polymorphisms and therefore were unlikely to be responsible, on their own, for the SUDY cases (Fig 1). Tables 1 and 2 compare the characteristics of all gene-positive and final gene-positive with gene-negative cases.

**Table 1:** Demographic characteristics of autopsy-negative cases.

	All gene-positive*	Gene-negative	P value
**Total**	27	101	
**Male/Female**	9/18 (33%/67%)	67/34 (66%/33%)	0.003
**Age at death in years (mean ± SD)**			
All	20.1 ± 12.7	21.5 ± 10.3	0.55
Males	16.2 ± 12.5	20 ± 10.3	0.31
Females	23.0 ± 13.1	24.3 ± 9.8	0.68
**Ethnicity**			
NZ European	21 (78%)	43 (42%)	0.001
Maori	0	31 (31%)	0.0009
Pacific Island	2 (7.3%)	10 (10%)	1.00
Chinese	2 (7.3%)	1 (1%)	0.11
Other	2 (7.3%)	11 (11%)	0.73
Not informed	0	5 (5%)	0.58
**Age groups**			
1–12	9 (33%)	14 (14%)	0.02
13–24	4 (15%)	47 (46%)	0.002
25–40	14 (52%)	40 (40%)	0.27
**Circumstances**			
Sleep	17 (63%)	46 (46%)	0.13
Exertion	3 (11%)	9 (9%)	0.71
Swimming	1(4%)	8 (8%)	0.68
Daytime	6 (22%)	38 (37%)	0.17
**Relevant medical history**			
Total	16/27 (59%)	32/88 (36%)	0.03
Seizures/epilepsy	14/16 (87%)	21/32 (65%)	0.11
**Relevant family history**			
Total	12/26 (46%)	19/72 (26%)	0.08
Seizures/epilepsy	2/12 (16%)	3/19 (16%)	1.00
SUDY/SUDI	7/12 (58%)	13/19 (68%)	0.70

SUDI—sudden unexplained death in infancy; SUDY—sudden unexplained death in the young

*All gene positive cases including those later downgraded to variants of unknown significance or polymorphisms

**Table 2:** Demographic characteristics of “final gene-positive” cases.

	Gene-positive	Gene-negative	P value
**Total**	13	101	
**Male/Female**	5/8 (38%/62%)	67/34 (66%/33%)	0.06
**Age at death in years (mean ± SD)**			
All	17.6 ± 14.2	21.5 ± 10.3	0.22
Males	14.5 ± 12.9	20 ± 10.3	0.09
Females	19.5 ± 15.5	24.3 ± 9.8	0.21
**Ethnicity**			
NZ European	11 (84%)	43 (42%)	0.004
Maori	0	31 (31%)	0.0009
Pacific Island	1 (8%)	10 (10%)	1.00
Chinese	1 (8%)	1 (1%)	0.21
Other	0	11 (11%)	0.36
Not informed	0	5 (5%)	1.00
**Age groups**			
1–12	7 (54%)	14 (14%)	0.004
13–24	1 (8%)	47 (46%)	0.007
25–40	5 (38%)	40 (40%)	1.00
**Circumstances**			
Sleep	6 (46%)	46 (46%)	1.00
Exertion	3 (23%)	9 (9%)	0.13
Swimming	0	8 (8%)	0.59
Daytime	4 (31%)	38 (37%)	0.76
**Relevant medical history**			
Total	6/13 (46%)	32/88 (36%)	0.48
Seizures/epilepsy	6/6 (100%)	21/32 (65%)	0.09
**Relevant family history**			
Total	7/13 (54%)	19/72 (26%)	0.04
Seizures/epilepsy	1/7 (14%)	3/19 (16%)	1.00
SUDY/SUDI	3/7 (43%)	13/19 (68%)	0.37

SUDI—sudden unexplained death in infancy; SUDY—sudden unexplained death in the young

New Zealand Europeans (Caucasians) were the majority in both groups; however the proportion of New Zealand Europeans in the gene-positive group was significantly higher (78% vs 42%, p = 0.001). Interestingly, Maori was the second most common ethnicity amongst gene-negative patients but none of the patients in the gene-positive group were Maori (31% vs 0, p = 0.0009). Other ethnicities did not differ between groups.

**Fig 2:**
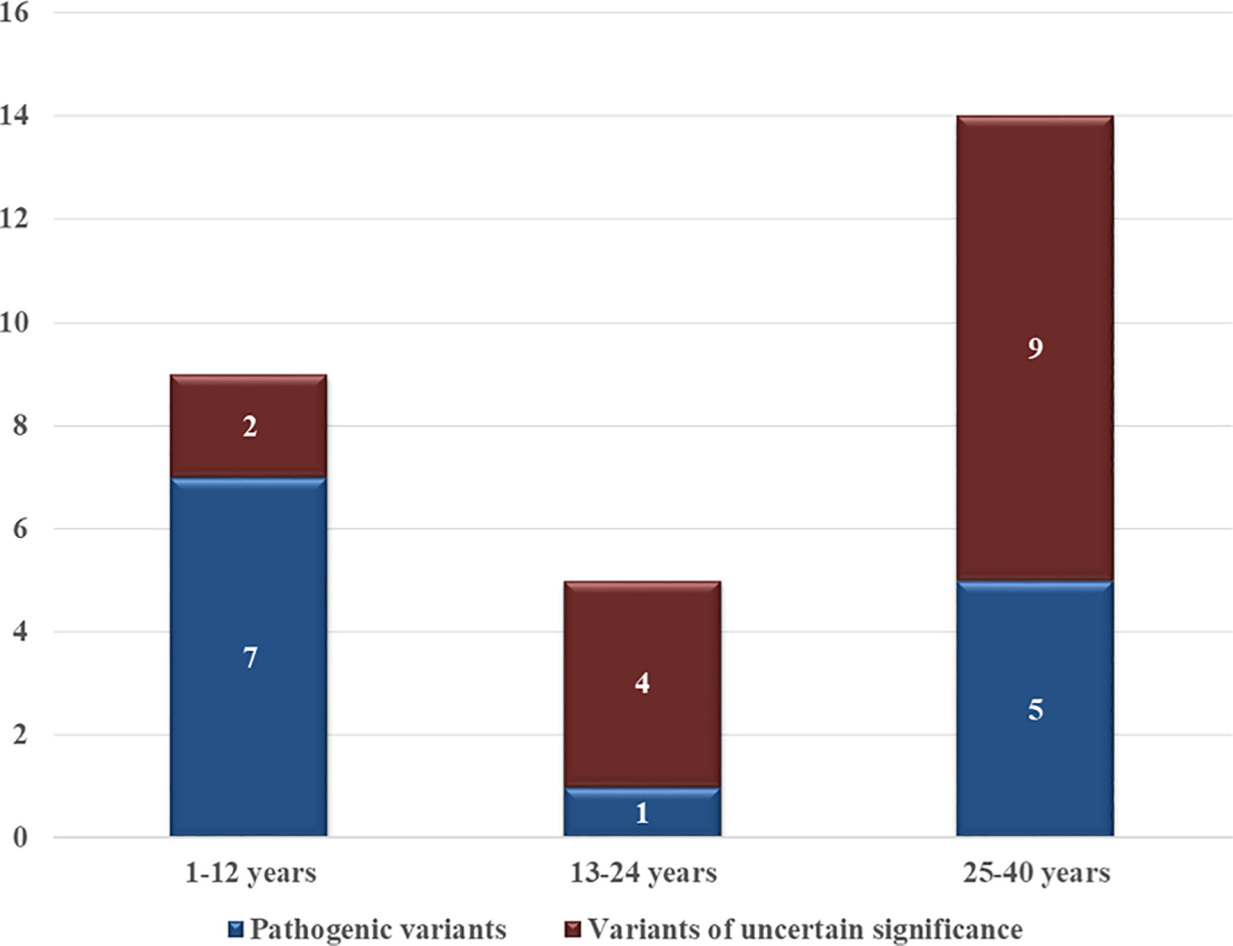
Distribution of pathogenic variants and variants of uncertain significance per age group.

The mean age of death did not differ between gene-positive and gene-negative cases. Males in the gene-positive group were younger but the difference did not reach statistical significance. The majority of gene-positive cases were in the older age group between 25–40 years when including all cases, but the likelihood of being gene-positive was higher between 1–12 years (33% vs 14%, p = 0.02), which increased for the “final gene-positive” group (54% vs 14%, p = 0.004, Fig 2). The opposite was seen for the age group between 13–24 years with a higher proportion being gene-negative (8% vs 46%, p = 0.007). No difference was seen for the older age group (25–40 years).

Circumstances of death did not differ between gene-positive and gene-negative cases. The majority of events happened during sleep (unwitnessed nocturnal deaths) in both groups, followed by daytime events. There were two deaths related to competitive sports in the gene-positive cohort (both during the activity) and four in the gene-negative cohort (two deaths during the activity and two immediately after).

Relevant past medical history was more prevalent amongst gene-positive cases (63% vs 36%, p = 0.01). History of seizures/epilepsy was the most prevalent past medical history reported in both groups, with no difference between gene-positive and gene-negative.

Relevant family history was more common amongst gene-positive cases but only significant when comparing the “final gene-positive” group with gene-negative (54% vs 26%, p = 0.04). History of seizures/epilepsy in family members, as well as SUDY and/or sudden unexplained death in infancy (SUDI) did not differ between groups.

### Analysis of genetic variants

Table 3 outlines the demographic characteristics, circumstances of death, previous medical history and family history of all gene-positive cases. Table 4 outlines each genetic variant, evidence of pathogenicity and likelihood of having caused SUDY. “Final gene-positive” subjects were highlighted in bold characters.

**Table 3:** Demographic characteristics, description of events, medical and family history of gene-positive cases.

Case	Sex	Age (y)	Ethnicity	Gene (s)	Circumstances of death	Medical history	Family history
1	F	1.0	Chinese	*SCN5A*	Sleep	No	No
**2**	**M**	**1.5**	**NZE**	***SCN5A***	**Sleep**	**No**	**SUDI**
				***SCN5A***			
**3**	**F**	**1.9**	**NZE**	***KCNH2***	**Daytime**	**Febrile seizures**	**No**
4	M	2.0	NZE	*SCN5A*	Sleep	Febrile seizures	SUDI and SUDY
				*SCN5A*			
**5**	**F**	**2.2**	**NZE**	***KCNQ1***	**Sleep**	**No**	**SUDI**
**6**	**F**	**3.6**	**NZE**	***SCN5A***	**Sleep**	**No**	**Syncope and SCA**
**7**	**M**	**9.9**	**NZE**	***KCNQ1***	**Daytime**	**Seizures and palpitations**	**No**
**8**	**M**	**12.5**	**NZE**	***KCNQ1***	**Exertion**	**Epilepsy**	**Syncope and epilepsy**
**9**	**M**	**12.6**	**NZE**	***KCNQ1***	**Exertion**	**No**	**No**
10	M	13.0	NZE	*KCNE2*	Swimming	Syncope	No
11	F	17.0	NZE	*KCNE1*	Daytime	Nocturnal seizures	SUDI
**12**	**F**	**19.5**	**NZE**	***KCNH2***	**Sleep**	**No**	**No**
13	F	19.9	NZE	*SCN5A*	Sleep	Nocturnal seizures, chromosomal abnormality*	No
14	F	25.0	American	*SCN5A*	Sleep	Epilepsy	Unknown
**15**	**F**	**25.2**	**Pacific Island**	***SCN5A***	**Sleep**	**Nocturnal seizures**	**Syncope**
16	M	25.8	NZE	*SCN5A* *SCN5A*	Sleep	Seizures and AF	No
**17**	**F**	**26.9**	**NZE**	***KCNH2***	**Sleep**	**Nocturnal seizures**	**No**
18	F	27.8	NZE	*KCNE2*	Sleep	No	No
19	F	29.4	NZE	*KCNH2*	Sleep	Palpitation with emotion	No
20	F	30.4	NZE	*SCN5A*	Daytime	Epilepsy	No
21	M	32.8	Pacific Island	*KCNH2*	Sleep	No	SUDY
22	F	33.6	NZE	*SCN5A*	Sleep	No	No
**23**	**M**	**36.1**	**Chinese**	***KCNH2*** ***SCN5A***	**Daytime**	**No**	**Syncope and SUDY**
24	F	36.6	Japanese	*KCNH2*	Sleep	Seizures	Epilepsy
25	F	36.9	NZE	*KCNE2*	Sleep	Nocturnal seizures	No
**26**	**F**	**37.6**	**NZE**	***KCNE1***	**Exertion**	**No**	**LQTS**
**27**	**F**	**39.56**	**NZE**	***KCNJ2***	**Daytime**	**Epilepsy**	**No**

AF: atrial fibrillation; LQTS: long QT syndrome; NZE: New Zealand European; SCA: sudden cardiac arrest; SUDI: sudden unexplained death in infancy; SUDY: sudden unexplained death in young.

*15p duplication

**Table 4:** Description of genetic variants and evidence of pathogenicity.

Case	Gene	Exon	Codon	Nucleotide change	Amino acid consequence	Description of the variant	Family members investigated (n)	Family screening supportive of diagnosis	Probability that the identified variant caused SUDY
1	*SCN5A*	20	1193	c.3578G>A	p.Arg1193Gln (R1193Q)	Previous reports[13]	3	No	Unlikely
**2**	***SCN5A***	**12**	**572**	**c.1715C>A**	**p.Ala572Asp (A572D)**	**Previous report**[13]	**5**	**No**	**Probable**
	***SCN5A***	**12**	**558**	**c.1673A>G**	**p.His558Arg (H558R)**	**Previous report**[13]			
**3**	***KCNH2***	**2**	**81**	**c.243G>C**	**p.Gln81His (Q81H)**	**Novel; reported by our group**[13]	**3**	**Yes**	**Probable**
4	*SCN5A*	12	546	c.1637A>G	p.Asp546Gly (D546G)	Novel; probably damaging/not tolerated on *in silico*[10, 11] analysis	9	No	Possible
	*SCN5A*	28	1950	c.5848T>C	p.Tyr1950His (Y1950H)	Novel; benign/tolerated on *in silico*[10, 11] analysis			
**5**	***KCNQ1***	**2**	**146**	**c.436G>A**	**p.Glu146Lys (E146K)**	**Previous report**[13]	**8**	**Partial**	**Probable**
**6**	***SCN5A***	**28**	**2004**	**c.6010T>C**	**p.Phe2004Leu (F2004L)**	**Previous reports**[13]	**6**	**Yes**	**Probable**
**7**	***KCNQ1***	**1**	**96**	**c.287C>G**	**p.Thr96Arg (T96R)**	**Novel; reported by our group**[13]	**3**	**Yes**	**Highly probable**
**8**	***KCNQ1***	**5**	**243**	**c.727C>T**	**p.Arg243Cys (R243C)**	**Previous reports and reported by our group**[13]	**5**	**Yes**	**Highly probable**
**9**	***KCNQ1***	**10**	**455**	**c.1363C>T**	**p.His455Tyr (H455Y)**	**Novel; reported by our group**[13]	**3**	**Yes**	**Highly probable**
10	*KCNE2*	2	8	c.22A>G	p.Thr8Ala (T8A)	Previous reports[13]	6	Partial	Possible
11	*KCNE1*	4	67	c.200G>A	pArg67His (R67H)	Previous report [13] and possibly damaging on *in silico* [10, 11]analysis	2	No	Possible
**12**	***KCNH2***	**9**	**749**	**2246delG**	p.Gly749Alafs*8 (G749Afs*8)	**Novel; frameshift; possibly damaging on *in silico***[10, 11]**analysis**	**11**	**Yes**	**Highly probable**
13	*SCN5A*	Intron 5		c.612-2A>G		Previous report [13]	9	No	Unlikely
14	*SCN5A*	6	216	c.647C>T	p.Ser216Leu (S216L)	Previous reports [13] and damaging/not tolerated on *in silico*[10, 11] analysis	0	No family screening	Possible
**15**	***SCN5A***	**Intron 15**		**c.2437-5C>A**		**Novel; likely pathogenic on splice-site analysis**	**17**	**Partial**	**Probable**
16	*SCN5A*	28	2004	c.6010T>C	p.Phe2004Leu (F2004L)	Previous reports[13]	9	No	Possible
	*SCN5A*	12	546	c.1673A>G	p.His558Arg (H558R)	Previous reports[13]			
**17**	***KCNH2***	**7**	**621**	**c.1861A>C**	**p.Ser621Arg (S621R)**	**Previous report**[13]	**3**	**Yes**	**Highly probable**
18	*KCNE2*	2	8	c.22A>G	p.Thr8Ala (T8A)	Previous reports[13]	3	No	Possible
19	*KCNH2*	4	262	c.784G>A	p.Gly262Ser (G262S)	Novel	9	No	Unlikely
20	*SCN5A*	20	1193	c.3578G>A	p.Arg1193Gln (R1193Q)	Previous reports[13]	4	No	Unlikely
21	*KCNH2*	12	968	c.2903C>T	p.Pro968Leu (P968L)	Previous reports[13]	3	No	Possible
22	*SCN5A*	28	2006	c.6016C>G	p.Pro2006Ala (P2006A)	Previous reports and reported by our group[13]	5	No	Possible
**23**	***KCNH2***	**7**	**561**	**c.1682C>T**	**p.Ala561Val (A561V)**	**Previous reports**[13]	**10**	**Yes**	**Highly probable**
	***SCN5A***	**Intron 15**		**c.2436+12G>A**		**Novel; benign/tolerated on *in silico***[10, 11] **analysis**			
24	*KCNH2*	6	502	c.1504A>C	p.Met502Leu (M502L)	Novel; tolerated on *in silico*[10, 11] analysis	0	No family screening	Uncertain
25	*KCNE2*	2	57	c.170T>C	p.lle57Thr (I57T)	Previous reports[13]	0	No family screening	Unlikely
**26**	***KCNE1***	**4**	**98**	**c.292C>T**	**p.Arg98Trp (R98W)**	**Previous reports**[13]	**10**	**Yes**	**Probable**
**27**	***KCNJ2***	**1**		**17q24.3del**		**Previous report**[14] **and reported by our group**[9]	**2**	**Yes**	**Highly probable**

Variants in the *SCN5A* gene were the most common (14/31, 45%), followed by *KCNH2* (7/31, 22%) and *KCNQ1* (4/31, 13%). Variants were less common in *KCNE2* (3/31, 10%), *KCNE1* (2/31, 7%) and *KCNJ2* (1/31, 3%). One case, a 12 year old boy with diabetes presented in detail elsewhere [13] was tested for *GPD1-L* and was positive, but we have not included his case in our current analysis.

#### SCN5A

*SCN5A* variants were the most common amongst both females (6/18, 33%) and males (4/9, 44%). Three males had two variants each in the *SCN5A* gene. Four out of the 13 “final gene-positive” cases had an SCN5A variant (31%). Two *SCN5A* variants were associated with a LQTS type 3 phenotype (cases 2 and 15) and 1 with a Brugada phenotype (case 6) according to previous reports, circumstances of death and family screening. One of the variants (case 23) was found in addition to a pathogenic *KCNH2* variant and was deemed benign as described below. The cases described below highlight particular issues our service has faced with defining pathogenicity.

Cases 1 and 20 had a variant (R1193Q) which has proven in vitro effect on channel function and has been linked to Brugada syndrome but is now recognised as a common polymorphism in Han Chinese. [15] Family screening was declined by one family of Chinese descent; in the other no phenotypic features of LQTS/Brugada were identified with cascade screening.

Case 4 had two novel variants (D546G and Y1950H), the former assessed as probably damaging/not tolerated on *in silico* analysis. Family screening did not contribute to establish or refute pathogenicity as one relative who was gene-positive for both variants had negative cardiac investigations including Ajmaline test. However, the deceased died during sleep and had a history of multiple febrile seizures, so a functional defect in the *SCN5A* gene cannot be ruled out as the cause of death.

Cases 6 and 16 had a variant (F2004L) linked to Brugada syndrome, [15] SUDI, [16] sudden death in women [17] and as a common polymorphism. [15] Case 6 had abnormal ante-mortem ECGs with features of Brugada syndrome and had documented VT/VF cardiac arrest. One of the parents declined genetic test but had a positive Ajmaline test, which in our view corroborates the pathogenicity of this variant in this family. Case 16 also carried the H558R polymorphism, which has been speculated to influence the functional phenotype of the F2004L variant. [18] The decedent had a previous history of epilepsy and atrial fibrillation. The H558R polymorphism has also been reported in association with lone atrial fibrillation. [19] Because of these characteristics we did not believe we could exclude a sodium channelopathy as the cause of death despite the fact that family screening did not contribute to elucidate pathogenicity of the variant in this family.

Case 13 had an intronic variant (c.612-2A>G) previously described in association with Brugada syndrome. [15] However, family screening identified two relatives with the same variant and negative cardiac investigations, including Ajmaline test, so we believe the genetic variant was not pathogenic in this family.

Case 23 had a novel intronic variant (c.2436+12G>A) which was not predicted to affect splicing and was deemed non-damaging/tolerated on *in silico* analysis. The other variant found in *KCNH2* has been described as likely pathogenic by previous reports. [15] Family screening revealed 10 gene-negative relatives, all with normal cardiac investigations. The decedent’s mother died of a cardiac arrest in the post-partum period, which is strongly suggestive of LQTS type 2.

#### KCNH2

Variants in *KCNH2* were found in 5/18 females (28%) and 2/9 males (22%). The results are shown in Table 4. Our main challenges here were related to small family size, but in some there was reasonably strong support by phenotype/genotype co-segregation. An example is case 12 who had a deletion resulting in a frameshift mutation (G749Afs*8). Six family members were found to have the same mutation with four having definite abnormal cardiac investigations.

#### KCNQ1

*KCNQ1* variants were more common in males (3/9, 33%), with only 1 female affected.

Case 5 had a variant (E146K) previously described as linked to LQTS. [15] Family screening revealed four gene-positive cases with normal cardiac investigations, all adults. One other child who died suddenly during infancy was found to have the same variant. We hypothesise that this might be a developmental variant associated with death during infancy/childhood which loses its pathogenicity in adulthood. [9] Cases 7, 8 and 9 all had variants previously described as pathogenic [15] and family cascade screening was supportive of the diagnosis of LQTS.

#### KCNE1

Two *KCNE1* variants were found in 2/18 females (11%) and no males.

Case 11 had a variant (R67H) previously reported as cause of LQTS, [15] but family screening did not support or refute the likelihood of pathogenicity. Case 26 had a variant (R98W) reported as pathogenic [15] and family screening revealed other gene-positive relatives with abnormal cardiac investigations.

#### KCNE2

*KCNE2* variants were found in 1/9 male (11%) and 2/18 females (11%). After our investigations we are not confident of pathogenicity in any of these cases, though we remain suspicious regarding two of them.

Case 10 was found to have a rare variant (T8A) previously reported as cause of drug-induced arrhythmia. [15] In addition, he had two previously reported common polymorphisms in *KCNH2* and *KCNE1* genes. At the time of death he was not taking any medications, but he died whilst swimming. It was unclear whether the combination of LQTS gene variants found in this male could have contributed to his death. Family screening revealed one gene-positive relative with borderline cardiac investigations as well one gene-negative with normal cardiac investigations.

Case 18 had the same variant as case 10. Cardiac investigations were suggestive of LQTS in two relatives, but the family declined genetic testing.

#### KCNJ2

Case 27 was found to have a deletion in 17q24.3, which encompasses the *KCNJ2* gene. Defects in *KCNJ2* have been linked to Andersen-Tawil Syndrome (ATS) [14] and novel mutations have been described in two families with LQTS without the ATS phenotype. [20] This female had a previous history of epilepsy and an abnormal ECG in life with a prolonged corrected QT on review of medical records. Her mother was found to carry the same deletion and has abnormal cardiac investigations. This case was reported previously our group. [10]

### Family screening

Screening was performed in 24 families (2 families were living overseas and 1 declined). A total of 148 family members were screened with either standard cardiac investigations alone (64/148, 44%) or in combination with genetic test (84/148, 56%). Amongst the ones that had genetic test, 42/84 (50%) were gene-positive. Definite LQTS or Brugada syndrome was diagnosed in 18/148 family members (12%), 13/18 (72%) females and 5/18 (28%) males. Seventeen families had genetic tests performed in both parents of the decedent. The genetic variant was maternally inherited in 13/17 families (76%) and paternally inherited in 4/17 (24%). Family cascade screening helped establish pathogenicity of the variant in 10/24 (41%) families. Therefore 10/27 variants (37%) were upgraded to “final gene positives” supported by family cascade screening.

## Discussion

New Zealand was the first country in the world to routinely provide molecular autopsy as part of a clinical cardiac genetic investigative service for SUDY. The first 26 months of this work demonstrated that the LQTS molecular diagnostic hit rate of 15% was somewhat lower in this population-based cohort than in a previous selected cohort. [21] Since that time, other centres around the world have incorporated genetic testing on the deceased into their investigations, although it remains far from routine. Countries such as the Netherlands and parts of the UK have pioneered cardiac investigation of the family, supported by genetic testing in living family members, as a means to achieve diagnosis in the deceased.

In 2008 members of the Trans-Tasman Response AGAinst sudden Death in the Young (TRAGADY) put forward guidelines to ensure standardization of autopsy practice in cases of SUDY. [22] This was endorsed locally by the Heart Foundation of New Zealand, Human Genetic Society of Australasia and the Royal Australasian College of Pathologists. The key features of this document were the performance of a thorough autopsy by an appropriately experienced pathologist, the early involvement of the family in the investigations, storage of tissue suitable for extraction of high quality DNA and referral to a cardiac genetic service. The essence of these guidelines has subsequently been adopted by the Heart Rhythm Society and the European Heart Rhythm Association. [23] This document formed a basis from which a recent bi-national study of young sudden death could progress. [7]

There remains however much to learn about in whom genetic testing is most valuable, whether it leads helpfully to a diagnosis in other family members and the relative benefits of the investigation of the family versus testing of the deceased. All of this occurs in the background of rapidly evolving and expanding genetic testing possibilities.

Of 365 sudden death cases referred to our multidisciplinary review panel, only 128, about one third, were finally put forward for molecular autopsy and family cardiac evaluation of first degree relatives. Cases were rejected for cardiac genetic evaluation on a number of grounds, with each case being different. Common examples include a positive toxicology result, evidence of significant co-morbidity, such as coronary artery disease or morbid obesity and hypertensive vascular changes, or florid myocarditis on histology. The constant scrutiny of autopsy reports, with ready access to second opinion, has led to an undoubted improvement in the quality of the reports, which at the start of the experience were often poor or incomplete. [24] Families of patients with another inherited heart disease at autopsy were also investigated, and genetic testing has often been done on the decedent, aimed at the disease concerned.

Of the 128 tested we found that one fifth of autopsy-negative SUDY cases carried a mutation or variant in the LQTS genes. From the start it has been a real challenge to define confidence in pathogenicity. Since 2006 much research has been done internationally, and many variants have been redefined from one level to another. There were no whole exome control populations at the start of this work. We decided to present our case series including those where a variant has been redefined because this is the real-life scenario that every service will have to come to terms with as such services develop. It is worthy of note that some variants may be benign or malignant depending on one’s ancestry. Rare variants in *SCN5A* are particularly challenging. In some cases such as the two year-old with nocturnal death and two variants (case 4), one is left with a sense that the channelopathy will have played a part, yet we cannot prove it. Perhaps they had a role like functional SNPs are postulated to do in SUDI, [18, 25] possibly with down-regulation of vulnerable channels by environmental factors [26] and other polymorphisms. [27] Overall, considering the 13 genetic variants ultimately classified as highly probably pathogenic, the minimum yield of molecular autopsy for this panel of genes was 10%. It is essential for the cardiac genetic service to have an open and frank, ongoing dialogue with the affected families and the forensic or coronial services. Most if not all families, once engaged with the service, appreciate the effort and attention that is given to them, and are able to accept a level of uncertainty when counseled appropriately.

We aimed to see if we could learn which cases are most likely to give a positive yield. Comparing variant-positive with negative cases, the proportion of females was significantly higher in the positive group. Caucasians were the majority in both groups, likely reflecting the population distribution in New Zealand. However, the proportion of Caucasians in the positive group was significantly higher and interestingly none of the cases in the positive cohort were of Maori ethnicity. According to a previous publication from our service, [8] the percentage of self-declared Maori amongst the population with LQTS in the Northern region of New Zealand was 22%, compared to 66% Caucasians. Considering that the Maori ethnic group accounts for approximately 15% of the total New Zealand population, it appears that Maori are proportionally represented amongst the living population with LQTS, but under-represented amongst the SUDY cohort. Further research is needed to establish the genetic profile and unique characteristics of the Maori population.

The majority of all variant-positive cases were in the older age group (25–40 years). However, if we only include the cases where we remain confident of pathogenicity, the proportion of final gene-positive cases in the youngest group (1–12 years) was significantly higher.

Death during sleep did not differ between variant-positive and negative cases. Two thirds of the variant positive cases died during sleep. The LQTS screen includes the *SCN5A* gene, linked to Brugada syndrome as well as LQTS type 3. These conditions classically cause sudden death in young adult males. Despite the majority of autopsy-negative SUDY cases being males, genetic testing in our cohort had a threefold higher yield of rare variants in females, particularly over 25. Interestingly, most cases were maternally inherited. Given these are autosomal dominant conditions; it is as yet unclear how this maternal inheritance might somehow confer increased malignancy of the channelopathy. This finding is, however, in keeping with previous studies [28, 29] and one potential explanation could be that epigenetic factors such as imprinting could be responsible for transmission distortion of LQTS genes.

Positive past medical history was significantly more prevalent in the variant-positive group. In a third of the cases sudden death was the first presentation of their condition. This is a recognised phenomenon, particularly with Brugada syndrome and LQTS type 3. A positive medical history was found in the remainder two thirds, implying that an opportunity to diagnose and prevent the death has been overlooked. Epilepsy and/or seizures, rather than standard cardiovascular symptoms such as palpitations were the most common medical history. Cerebral hypoxia as a consequence of low cardiac output causes seizures. As previously reported in the literature, including by our group, [30] incorrect diagnosis of epilepsy is common in patients with LQTS. This present report suggests that awareness of this fact still needs to be raised in order to avoid these tragic outcomes.

Mutations and rare variants in *SCN5A* and *KCNH2* were the most frequent, which is in keeping with a higher risk of sudden death at younger ages for LQTS types 2 and 3. [31] All but one case of *SCN5A* gene positive died during sleep, as expected.

Tester et al reported a high incidence of *RyR2* mutations (linked to CPVT) in their SUDY cohort, [5] and subsequently the standard molecular autopsy recommended including *RyR2* in the panel. [23] This test was not available to us consistently over the reported time period. If we had we would have expected more deaths during exercise or activity to be present in our gene-positive cohort.

Relevant family history was found in about half of the cases in which family history could be obtained. There was a high prevalence of SUDY and/or SUDI and syncope was the second most common diagnosis. None of the families had a previous diagnosis of LQTS or had been investigated for an inherited cardiac disease at any time prior to the demise of their relative. A high prevalence of positive family history of SUDY without prior diagnosis has been described previously [32, 33] and unfortunately this is still a reality. It confirms that awareness of the possibility of an inherited cardiac disease following SUDY is still lacking for the majority of medical professionals who deal with these families.

Family screening identified 18 individuals with LQTS and Brugada syndrome who are now being appropriately managed. It also helped support the diagnosis of LQTS/Brugada through co-segregation in just over a third of cases.

The high frequency of novel variants found by molecular autopsy means that evaluation of family members is often required to confirm pathogenicity. We consider engagement of the family to be an essential part of this autopsy investigative process, as originally recommended by the TRAGADY document. As in our previous experience with the investigation of SIDS we do not recommend molecular autopsy being performed as a stand-alone test. [9] Furthermore, the value of the test is of course greatly enhanced by its potential use in family cascade screening.

The next phase of development of the molecular autopsy will be multiple gene panels or whole exome/genome sequencing. Recent population studies using such technology have led to a number of previously described likely pathogenic mutations being reclassified as common polymorphisms. [34, 35] This new information led to a reduction in the number of cases in our initial cohort with a highly probable or probable diagnosis of LQTS or Brugada syndrome, as some of the variants initially found were later reclassified. These findings enhance the importance of family engagement, genetic counseling, and subsequent co-segregation studies to evaluate novel variants within families.

The aim of our work is to prevent sudden deaths through identification of those at risk in the community. We have shown previously that aggressive family cascade screening of all probands, dead or alive, can lead to effective identification of LQTS in the community. Half of the anticipated 1 in 2000 cases of LQTS have already been identified in parts of New Zealand. [8] The approach described here is part of that initiative.

### Limitations

The molecular autopsy was limited to assessment of variants in the LQTS genes and only *SCN5A* for Brugada. This is a practical and fiscal issue, faced by many countries across the world. We do not have routine access to *in-vitro* assessment of genetic variants, and a limitation of this study and the majority of studies that deal with genetic mutations is the difficulty is attributing pathogenicity to a rare variant. This will only become harder as wider genetic panels reveal more variants of uncertain significance. [36] We tried to use as many indicators of pathogenicity as possible, but a definite correlation between a genetic variant and SUDY is not always straightforward and new information can change a genetic variant from likely pathogenic to a common polymorphism with definite implications for the families involved. This is why family co-segregation is so important and is still ongoing for some of the families in our cohort.

## Conclusion

The LQTS molecular autopsy contributed to a likely diagnosis in one fifth of SUDY cases, although this was only confirmed in half of the cases by family screening and further literature support. *SCN5A* and *KCNH2* variants predominated. Whites, adult females and children aged 1–12 had the highest yield of a positive genetic test in our cohort. There was also evidence that mutations inherited maternally are over represented. Of those with a genetic diagnosis, past medical history of syncope, seizures and/or epilepsy was common as well as family history of sudden death. Family screening proved valuable in detecting a significant number of affected family members and in providing additional supportive evidence of pathogenicity of some of the rare variants encountered.
